# Visualization of regional tau deposits using ^3^H-THK5117 in Alzheimer brain tissue

**DOI:** 10.1186/s40478-015-0220-4

**Published:** 2015-07-02

**Authors:** Laetitia Lemoine, Laure Saint-Aubert, Amelia Marutle, Gunnar Antoni, Jonas P Eriksson, Bernardino Ghetti, Nobuyuki Okamura, Inger Nennesmo, Per-Göran Gillberg, Agneta Nordberg

**Affiliations:** Department of Neurobiology, Care Sciences and Society, Center for Alzheimer Research, Division of Translational Alzheimer Neurobiology, Karolinska Institutet, Novum, 5th floor, Stockholm, S-14157 Sweden; Preclinical PET Platform, Department of Medicinal Chemistry, Faculty of Pharmacy, Uppsala University, Uppsala, Sweden; PET Centre, Centre for Medical Imaging, Uppsala University Hospital, Uppsala, Sweden; Department of Pathology & Laboratory Medicine, Indiana University School of Medicine, Indianapolis, IN USA; Department of Pharmacology, Tohoku University School of Medicine, Sendai, Japan; Department of Pathology, Karolinska University Hospital, Stockholm, Sweden; Department of Geriatric Medicine, Karolinska University Hospital Huddinge, Stockholm, Sweden

**Keywords:** Alzheimer’s disease, Tau pathology, THK5117, Imaging biomarker, Autopsy brain, Autoradiography

## Abstract

**Introduction:**

The accumulation of neurofibrillary tangles, composed of aggregated hyperphosphorylated tau protein, starts spreading early in specific regions in the course of Alzheimer’s disease (AD), correlating with the progression of memory dysfunction. The non-invasive imaging of tau could therefore facilitate the early diagnosis of AD, differentiate it from other dementing disorders and allow evaluation of tau immunization therapy outcomes. In this study we characterized the *in vitro* binding properties of THK5117, a tentative radiotracer for positron emission tomography (PET) imaging of tau brain deposits.

**Results:**

Saturation and competition binding studies of ^3^H-THK5117 in post-mortem AD brain tissue showed the presence of multiple binding sites. THK5117 binding was significantly higher in hippocampal (p < 0.001) and temporal (p < 0.01) tissue homogenates in AD compared to controls. Autoradiography studies with ^3^H-THK5117 was performed on large frozen brain sections from three AD cases who had been followed clinically and earlier undergone *in vivo*^18^F-FDG PET investigations. The three AD cases showed distinct differences in regional THK5117 binding that were also observed in tau immunohistopathology as well as in clinical presentation. A negative correlation between *in vivo*^18^F-FDG PET and *in vitro*^3^H-THK5117 autoradiography was observed in two of the three AD cases.

**Conclusions:**

This study supports that new tau PET tracers will provide further understanding on the role of tau pathology in the diversity of the clinical presentation in AD.

**Electronic supplementary material:**

The online version of this article (doi:10.1186/s40478-015-0220-4) contains supplementary material, which is available to authorized users.

## Introduction

Alzheimer’s disease (AD) is the most common form of dementia among the elderly. The two pathological hallmarks of AD are fibrillar amyloid beta (Aβ) deposition in the brain in the form of senile plaques (SPs), and accumulation of hyperphosphorylated tau protein in the form of neurofibrillary tangles (NFTs). Although the relationship between SPs and NFTs remains unclear, both appear to precede the onset of clinical symptoms of AD [1]. Several biomarkers have been developed to target the pathological features *in vivo* in order to help the clinical diagnosis of AD, showing increased reliability with time. Quantification of Aβ and tau from cerebrospinal fluid (CSF) appears consistent with quantification of the lesions [2, 3]. However this invasive method is indirect, as the relationship between CSF biomarkers and AD lesions is still unclear [4]. Positron emission tomography (PET) imaging has been developed over recent decades and is now a very important tool for diagnosing AD, with particular regard to *in vivo* assessment of the cerebral amyloid load. However, the pathophysiological link between formation of the amyloid plaques and neurodegeneration is still unknown, and amyloid-targeting drug therapies have so far failed to show conclusive results.

In order to visualize and increase our understanding of the time course of NFT deposition during the development of AD and other dementia disorders, there is currently an attempt to develop PET tracers that are specific for tau protein [5]. Postmortem studies on patients with confirmed AD have shown a correlation between NFTs (but not SPs) and cognitive impairment [6–8]. Since tau aggregates are mainly intracellular, the radiotracer has to cross the blood–brain barrier as well as the cell membrane. Moreover, the tau ligand needs to be very specific for NFTs; in some regions of AD affected brains, amyloid plaques and NFTs occur together, although the occurrence of amyloid plaques is 5–20 times greater than that of NFTs [9]. The PET tracer ^18^F-FDDNP, initially introduced as an Aβ PET tracer, also binds to NFTs [10]. A negative correlation between ^18^F-FDDNP binding and episodic memory has been reported in patients with AD and patients with mild cognitive impairment (MCI) [11], but this tracer showed limitations with low levels of specific binding [12]. Recently, several PET tau tracer candidates from three different chemical classes have been developed, including the THK analogues ^18^F-THK523, ^18^F-THK5105, and ^18^F-THK5117, as well as the ligands ^11^C-PBB3, ^18^F-T808 and ^18^F-T807 [5, 13]. Clinical studies with ^18^F-THK5117, ^11^C-PBB3, and ^18^F-T807 are currently in progress [14–17].

In this study, we characterized the *in vitro* binding properties of THK5117 in large frozen sections from autopsied AD brains, and compared its binding pattern with measured tau histopathology as well as with previous *in vivo* measurement of ^18^F-FDG and ^11^C-Pittsburgh compound B (PIB) PET imaging in the same patients.

In this study, we focused on the ^18^F-THK5117 tracer that was reported to have good binding properties in AD tissue [17]. Here we aimed at investigating in greater details the THK5117 binding using large frozen sections from autopsied AD brains, and compared its binding pattern with measured tau histopathology as well as with previous *in vivo* measurement of ^18^F-FDG PET imaging in the same patients.

## Materials and methods

### Chemicals

1-Fluoro-3-((2-(4-([^3^H]methylamino)phenyl)quinolin-6-yl)oxy)propan-2-ol (^3^H-THK5117; specific activity, SA 2.2 GBq/μmol) and N-methyl-[^3^H]2-(4′-methylaminophenyl)-6 hydroxybenzothiazole (Pittsburgh compound B, ^3^H-PIB; SA 2.3 GBq/μmol) were custom synthesized by Quotient Bioresearch (Cardiff, UK). Unlabeled THK5117 was synthesized by Tanabe R&D Service (Osaka, Japan) and by Quotient Bioresearch (Cardiff, UK). 2-(1-{6-[(2-fluoroethyl) (methyl)amino]-2-naphthyl}ethylidene)malononitrile (FDDNP) was a kind gift from Dr Jorge R. Barrio (UCLA, USA). BTA-1 2-(4-methylaminophenyl)benzothiazole was purchased from Sigma Aldrich (Sweden).

### *In vitro* binding assays

Human brain tissues from eight Alzheimer patients and eight controls were obtained from the Netherlands Brain Bank (demographic details in Table 1), and homogenized in PBS containing 0.1 % BSA and protease/phosphatase inhibitor.

**Table 1:** Clinical information for binding assay studies

	Age (Years)	Gender (M/F)	ApoE (E/E)	Braak stage	Postmortem delay (hours)
Alzheimer cases	***59***	***F***	***4/4***	***5***	***4***
	***61***	***M***	***3/3***	***5***	***4***
	66	F	4/3	5	7
	70	M	4/4	4	4
	***75***	***F***	***4/4***	***5***	***6***
	***78***	***M***	***4/4***	***5***	***7***
	81	F	4/3	5	6
	85	F	3/3	4	6
	**72 ± 9**	**3 M/5 F**	**2 E3**	**4-5**	**5 ± 1**
			**6 E4**		
Controls	50	F	3/3	1	17
	62	M	3/3	1	7
	71	F	3/2	1	7
	77	F	3/3	1	3
	78	M	3/3	1	7
	79	M	3/3	2	9
	81	M	3/3	2	8
	84	F	3/3	1	4
	**73 ± 11**	**4 M/4 F**	**8 E3**	**1-2**	**8 ± 4**
			**0 E4**		

The table presents information for the binding studies cases from which homogenates have been extracted. The age at death, gender, apolipoprotein E (ApoE) genotype, Braak stage at cerebral pathological assessment, and post-mortem delay are given for each patient included in the study

M = male; F = Female. Temporal lobes from AD cases mentioned in bold have been used for the saturation and competition studies, as well as the hippocampus from the 75 years-old AD case

Optimal conditions for the binding assay study were determined in preliminary experiments by varying the time of incubation (1 h, 2 h or 3 h), the tissue concentration (100 μg/mL, 200 μg/mL, 500 μg/mL or 750 μg/mL) and the buffer (PBS, PBS + 0.1%BSA or Tris buffer). 

Saturation binding assays were carried out with incubation of temporal cortex and hippocampus homogenates (0.1 mg tissue) with ^3^H-THK5117 (0.04-270 nM) for 2 h at room temperature. Non-specific binding was determined with 1 μM THK5117. The binding reaction was terminated by filtering through glass fiber filters (pre-soaked for 3 h in 0.3 % polyethylenimine), and rinsing three times with cold binding buffer, and counted with a beta scintillation counter (Beckman Coulter LS6500). The results were analyzed with GraphPad Prism software (version 5.0f for Mac OS X, GraphPad Software, San Diego California USA). The equilibrium dissociation constant (Kd) and maximum number of binding sites (Bmax) were then determined.

The specificity and selectivity of ^3^H-THK5117 binding was determined by incubating homogenates (0.1 mg tissue) from Alzheimer’s patients’ temporal cortex and hippocampus with ^3^H-THK5117 (3 nM), in the presence of THK5117 (10^−5^ -10^−13^ M), FDDNP (10^−6^ -10^−14^ M) or BTA-1 (10^−5^ -10^−13^ M). The results were analyzed with GraphPad Prism software, and the resulting inhibitory constants (Ki) were determined.

Regional binding studies with ^3^H-THK5117 were carried out on the brain homogenates from temporal cortex, frontal lobe, parietal lobe, caudate nucleus, hippocampus and cerebellum that were incubated with a single concentration of ^3^H-THK5117 (3 nM). Non-specific binding was determined with 1 μM of THK5117. Comparisons between AD and control tissues were made using Mann–Whitney non-parametrical tests, with a threshold for significance of p < 0.05.

### Autoradiography binding studies in Alzheimer’s disease and control autopsy brain tissue

Postmortem brain tissue for autoradiography studies was obtained from three patients with AD that all three had been clinically followed (by A.N.) at the Department of Geriatric Medicine, Karolinska University Hospital, Huddinge, Sweden. The left hemisphere of each brain was frozen in large block sections. The right hemispheres were fixed in formaldehyde, brain tissue was sampled according to the BrainNet Europe protocol and histopathological investigations were undertaken (by I.N.) to confirm AD pathology (see Additional file 1).

AD case one was referred for memory assessment at 49 years of age due to complaint of progressive memory impairment over 2 years. The patient was diagnosed with AD, and received galantamine (cholinesterase inhibitor) and memantine (NMDA antagonist) treatment. The patient was followed clinically and underwent two ^18^F-FDG PET at MMSE 18/30 and 12/30, respectively. After 4 years in a nursing home, the patient died at the age of 60, and autopsy (post-mortem delay of 25 h) confirmed AD pathology, with Braak stages V-VI. General atrophy of the gyri was observed, especially the frontal lobe, and severe atrophy of the amygdala and the anterior part of the hippocampus.

AD case two was referred at the age of 71 with a 7-year history of memory complaint. Neuropsychological testing showed impairment in several cognitive domains with a MMSE score of 26/30, and CSF biomarkers were pathological. The patient was diagnosed with AD, treated with phenserine and rivastigmine (cholinesterase inhibitors) and underwent ^18^F-FDG PET. The patient died aged 79 years, after being in a nursing home for 2 years, and autopsy (post-mortem delay of 16 h) confirmed AD with at least Braak stage V. The brain presented with generally slightly smaller gyri than normal, and atrophic anterior part of the hippocampus.

AD case three was referred at 70 years of age with a 7-year history of memory complaint. Neuropsychological testing showed disturbances in episodic memory, spatial orientation, attention, and MMSE was at 26/30. CSF biomarkers were pathological, and MRI showed frontal atrophy. The patient was diagnosed with AD, treated with rivastigmine (anticholinesterase inhibitor) and memantine (NMDA antagonist, and underwent three ^18^F-FDG PET examinations at 70, 72 and 75 years of age. The patient died aged 81 years after four years in a nursing home, and autopsy (post-mortem delay of 17 h) confirmed Braak stages V-VI with slight atrophy of the frontal lobe.

Control case was a 76-year-old female, with a postmortem delay of 4 h and Braak stage I, obtained from the Neuropathology of Dementia Laboratory (Indiana University School of Medicine, Indianapolis, USA).

Frozen brain sections (80 μM) from the left hemispheres of the three AD cases and the healthy control case were cut with a cryostat at −20 °C and thaw-mounted on chrome alum gelatin-subbed slides following a previously described protocol [18].

For each case, frozen sections were dried, pre-incubated for 10 min with PBS + 0.1%BSA, and were then incubated for one hour with 4 nM ^3^H-THK5117. The non-specific binding assay was performed on adjacent sections in the presence of 10 μM THK5117. After incubation, sections were washed 3 times for 5 min with cold incubation buffer, dipped into cold distilled water, and allowed to dry for at least 24 h. A Fujifilm BAS-2500 phosphor imaging plate was then exposed to the section, along with a tritium standard, for three days (Larodan Fine Chemical AB, Malmö, Sweden) before being scanned by a phosphoimager Fujifilm BAS-2500.

Binding of the tracer was quantitatively assessed using MultiGauge Fujifilm software (V3.0). Two independent raters manually segmented different regions of interest, as defined by Duvernoy in “The Human Hippocampus” [19], resulting in regional mean uptake values for each segment. Inter-rater agreement was assessed for all uptake values using a Bland-Altman plot, with limits of agreement set to 95 %. When agreement was satisfactory, regional uptake values from both raters were averaged for subsequent analyses.

Autoradiography with enantiomerically pure (*S*)-^18^F-THK5117 was also performed on adjacent sections for each case. The sections were pre-incubated for 10 min with PBS + 0.1%BSA and incubated for 15 min with 0.5 MBq/mL (*S*)-^18^F-THK5117. Non-specific binding was determined in adjacent sections by adding 10 μM THK5117. After incubation, sections were washed three times 5 min with cold buffer and then dipped into 50 % v/v cold distilled water and ethanol before drying in an incubator at 37 °C. A phosphor imaging plate was exposed to the sections overnight, before scanning with a phosphoimager (Cyclone plus, Perkin Elmer).

### Autoradiography and immunostaining on small paraffin AD brain sections

Routine pathology with AT8 has been performed on anterior and posterior hippocampus paraffin sections from the right hemisphere of the three AD cases. Autoradiographies with ^3^H-THK5117 on adjacent sections have been performed after deparafinization following the same protocol as cited above.

A paraffin section from hippocampus of a patient with confirmed AD (70 years old at death, Braak VI, from the Netherlands brain bank) were quenched and stained with THK5117 following the procedure used by Okamura and colleagues [17, 20] for autofluorescence studies. An adjacent section was labeled with AT8 anti-tau and assessed using the EnVision + System HRP (DAB) (Dako Denmark) detection method.

### *In vivo* imaging

Image Acquisition: PET Data: All ^18^F-FDG PET investigations were carried out at the Uppsala PET centre using ECAT EXACT HR+ scanners (Siemens/CTI). The mean injected dose was 229 ± 49 MBq. Acquisition was performed during 45 min after injection. All reconstructed frames were realigned to correct for patient motion during each PET scan. The sum image from 25 to 45 min was used for analyses.

MRI Data: Case two and case three underwent a structural T1 MRI sequence using a 3 T Siemens Trio scanner. Images were acquired with a slice thickness of 1.5 mm and were reconstructed to 1.0 x 1.0 x 1.0 mm isometric voxels.

Regional quantification of ^18^F-FDG PET images for correlation with *in vitro* autoradiography: In order to compare the *in vitro* quantitative autoradiography binding measurements with the *in vivo*^18^F-FDG PET images, the coronal section of the T1 MRI judged to correspond best to the relevant autoradiography section was manually segmented by two independent raters. The atlas used for segmentation of autoradiographies (see above [19]) was used to define regions of interest on the T1 section. Mean uptake values were calculated for each of these regions on the registered ^18^F-FDG PET images.

Inter-rater agreement was assessed for all resulting regional uptake values using a Bland-Altman plot, with limits of agreement set to 95 %. When agreement was satisfactory, regional uptake values from both raters were averaged for subsequent analyses.

The relationship between ^18^F-FDG regional uptake values measured by PET and the *in vitro* binding results from autoradiography was assessed using a Spearman correlation test.

## Results

### THK5117 multiple binding sites

**Fig. 1:**
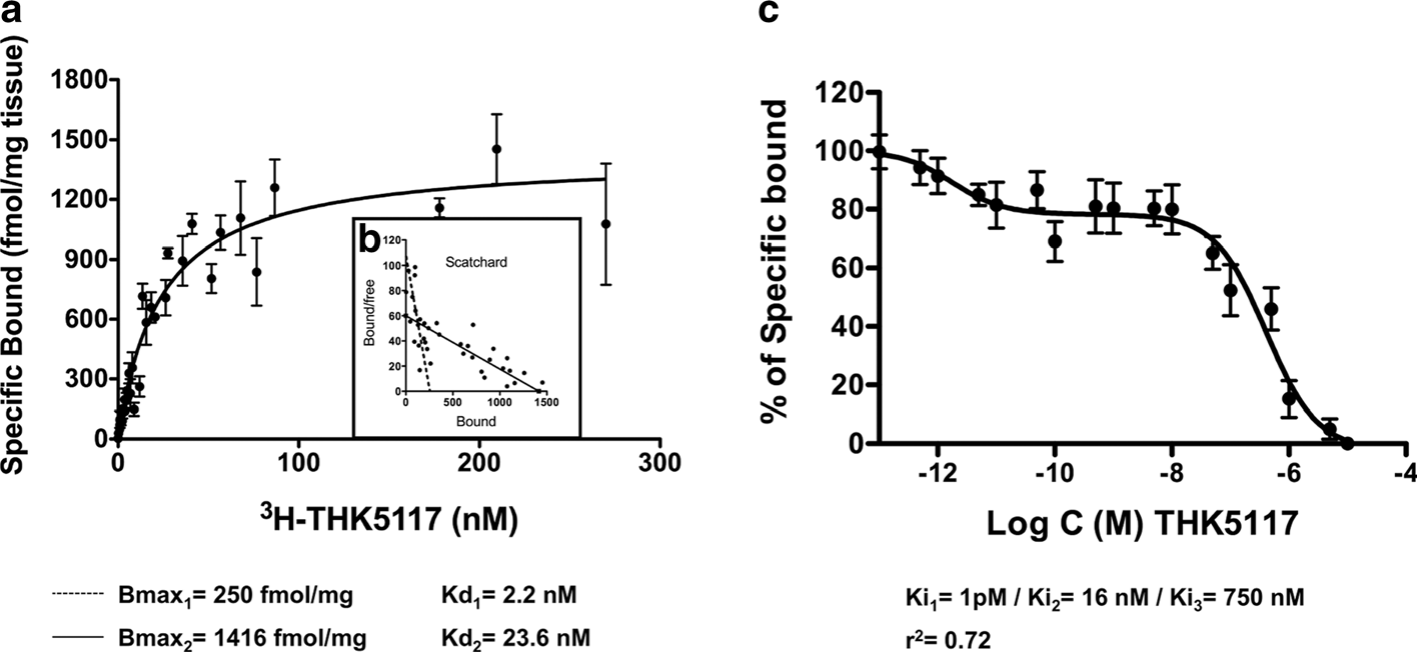
Saturation binding curve (**a**) and Scatchard plot (**b**) for ^3^H-THK5117 (0.04-270 nM) in temporal lobe brain homogenates from four patients with AD. The solid regression line was determined by GraphPad Prism software and corresponds to the low affinity site. The dotted regression line was determined manually and corresponds to the high affinity site. The corresponding Kd and Bmax values are given below the curve. (**c**) Competition binding curve between THK5117 (10^−13^-10^−5^ M) and ^3^H-THK5117 (3 nM) in temporal lobe brain homogenates from four patients with AD. Analyses from non-linear regression using a least square ordinary fit in GraphPad Prism software showed three binding sites. Ki: inhibitory constant. r^2^ = regression coefficient

Saturation binding assay performed with increasing concentrations of ^3^H-THK5117 (0.04-270 nM) in post-mortem AD temporal brain tissue homogenates showed saturation with a Bmax of 1416 fmol/mg and Kd_2_ of 24nM (Fig. 1a). A second binding site (Bmax 250fmol/mg and Kd_1_ of 2.2 nM) was determined manually from the Scatchard plot (Fig. 1b). Saturation binding data from the temporal cortex were averaged from all seven experiments.

Saturation binding in the hippocampus (one Alzheimer brain) revealed two binding sites, with Kd values of 3.1 nM and 34 nM and Bmax values of 250 fmol/mg and 1226 fmol/mg, respectively (Additional file 2).

Competition studies with increasing concentration of unlabeled THK5117 (10^−5^-10^−13^ M) and ^3^H-THK5117 (3 nM) in AD temporal cortices (Fig. 1c) showed two binding sites (Ki_2_ = 16 nM, Ki_3_ = 750 nM). Results were averaged from ten competition experiments. Setting the constraints in the statistical model to >100 for the start of the curve indicated the presence of a potential third site (Ki_1_ = 1 pM).

### Selectivity of THK5117

Increasing concentrations of FDDNP showed a trend for competition with ^3^H-THK5117 for a super-high affinity binding site (picomolar range) and a low affinity site (micromolar range; Additional file 3a). No competition was observed neither between ^3^H-THK5117 and BTA-1 nor between ^3^H-PIB and unlabeled THK5117 (Additional file 3b).

### Regional binding distribution of ^3^H-THK5117 in tissue homogenates

**Fig. 2:**
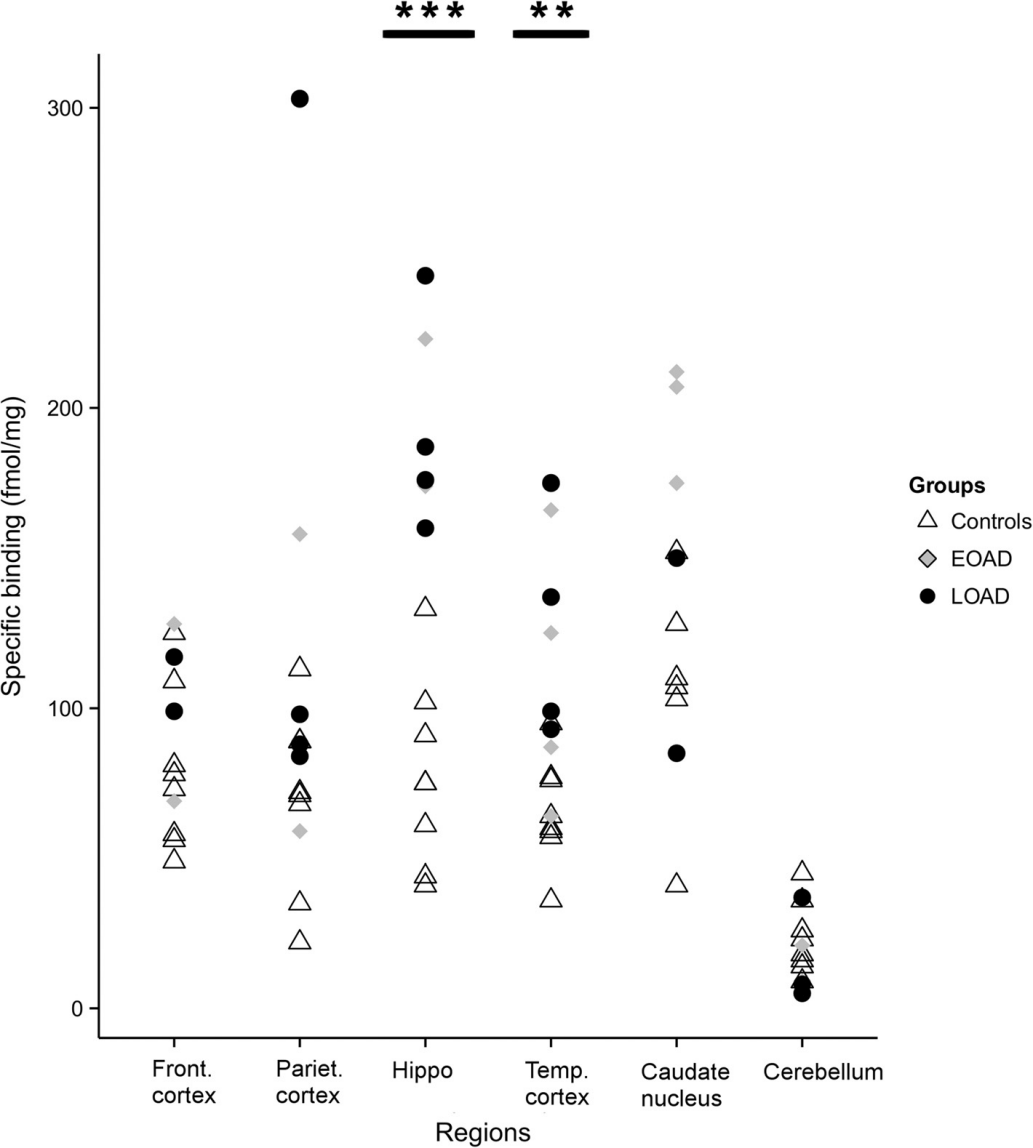
Regional binding distribution of ^3^H-THK5117 in homogenates from eight patients with AD and eight healthy controls. EOAD = patients with Early onset Alzheimer’s Disease, LOAD = patients with Late Onset Alzheimer’s Disease, Front. =Frontal, Pariet. = Parietal, Hippo = Hippocampus, Temp. = Temporal. Statistical significance is shown for group comparison between patients (EOAD + LOAD) and controls. ** p < 0.01; ***p < 0.001

The regional binding distribution of ^3^H-THK5117 in the AD cases was highest in the hippocampus (198 ± 67 fmol/mg), followed by the caudate nucleus (198 ± 30 fmol/mg), the temporal lobe (110 ± 47 fmol/mg) and the parietal lobe (105 ± 46 fmol/mg), and was very low in the cerebellum (Fig. 2). Statistical group comparisons revealed a significantly higher binding in the AD than in the controls in the hippocampus (p < 0.001) and the temporal region (p = 0.005) (Fig. 2).

Binding in caudate nucleus was found higher in the three patients with early onset AD (EOAD<65 years old) cases than in the three with late onset AD (LOAD>65 years old).

### Regional distribution of ^3^H-THK5117 evaluated by autoradiography

**Fig. 3:**
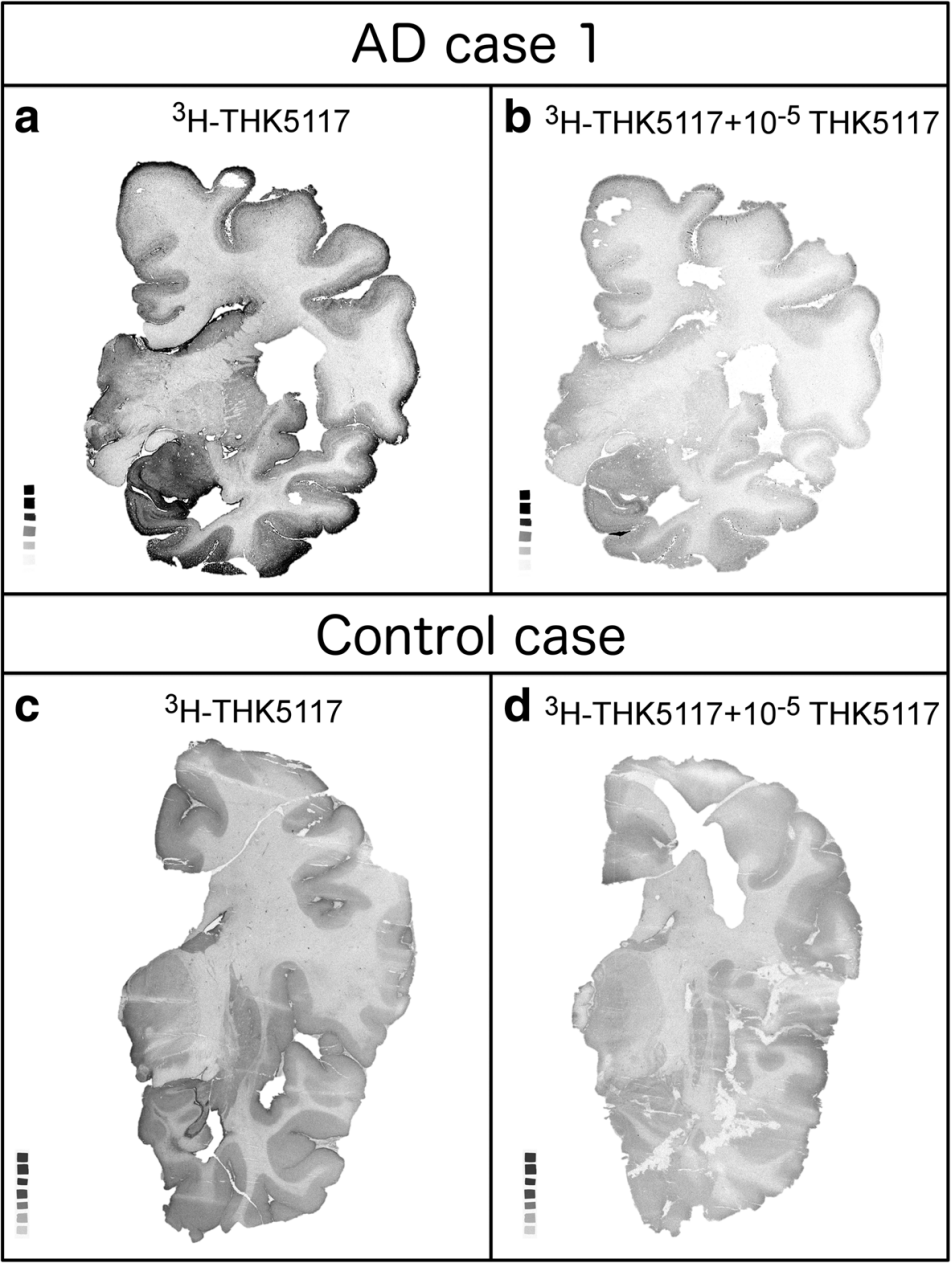
Autoradiography results from adjacent left frozen hemisphere sections from AD case one and the control case. (**a**) ^3^H-THK5117 in AD case one. (**b**) ^3^H-THK5117 + 10^−5^ M of THK5117 in AD case one. (**c**) ^3^H-THK5117 in control case. (**d**) ^3^H-THK5117 + 10^−5^ M of THK5117 in control case

The total and non-specific binding autoradiograms for AD case one and the control case are shown in Fig. 3, while autoradiograms for AD case two and three are shown in Additional file 4. From those autoradiograms, fourteen regions of interest could be defined for case one, and twelve for case two and three. Quantitative results are presented in Table 2 for each case.

**Table 2:** Clinical information and regional specific binding of ^3^H-THK5117 on autoradiography sections from the three Alzheimer cases

Demographic data			
Demography	AD Case 1	AD Case 2	AD Case 3
Onset	EOAD	LOAD	LOAD
Gender (M/F)	F	F	M
ApoE alleles (E/E)	4/4	4/4	4/4
MMSE at time of last PET	12	26	25
Age at death (years)	60	79	81
Time between last PET and death (years)	7	7	5
^3^H-THK5117 specific binding in regions of interest (fmol/mg)			
Regions of interest	AD Case 1	AD Case 2	AD Case 3
Hippocampus	693	171	112
Entorhinal cortex	713	126	53
Fusiform gyrus	585	189	52
Inferior temporal gyrus	764	125	59
Middle temporal gyrus	495	140	48
Superior temporal gyrus	363	114	53
Insular cortex	423	68	50
Postcentral gyrus	312	77	75
Precentral gyrus	310	32	102
Middle frontal gyrus	276	55	56
Superior frontal gyrus	344	57	114
Cingulate gyrus	292	120	110
Putamen	242	NA	NA
Thalamus	349	NA	NA
Amygdala	507	NA	NA

^3^H-THK5117 binding values were obtained by subtracting the nonspecific binding values from the total binding values. Regions of interest were defined for each case by manual segmentation of the section

AD = Alzheimer’s Disease; EOAD = Early Onset Alzheimer’s Disease; LOAD = Late Onset Alzheimer’s Disease; M = male; F = Female; NA = not available

Inter-rater agreement for manual segmentation was within the 95 % limit for agreement.

Specific ^3^H-THK5117 binding in case one was intense in the hippocampus, the temporal cortex and the frontal cortex. ^3^H-THK5117 binding in AD case two and three was less intense than in AD case one; specific binding was high in the hippocampus and the cingulate for AD case two, and high in the hippocampus and the frontal cortex for AD case three. Furthermore, the binding intensities differed within a single brain region, as illustrated by the values in temporal gyri for case one. Low and regional binding was found in the control case compared to the three AD cases (Fig. 3).

As shown in Table 2, there were large differences among the three patients with respect to ^3^H-THK5117 binding in the regions studied. Regional ^3^H-THK5117 binding was highest in case one and lowest in case three. The differences between the three cases were greatest in the entorhinal cortex, inferior temporal cortex, middle temporal cortex and fusiform gyrus. In all three cases, hippocampus was one of the regions with highest ^3^H-THK5117 binding.

Autoradiography results for (*S*)- ^18^F-THK5117 indicated a similar binding pattern to that for ^3^H-THK5117 (Additional file 5).

### Validation of the selectivity of THK5117 towards TAU

**Fig. 4:**
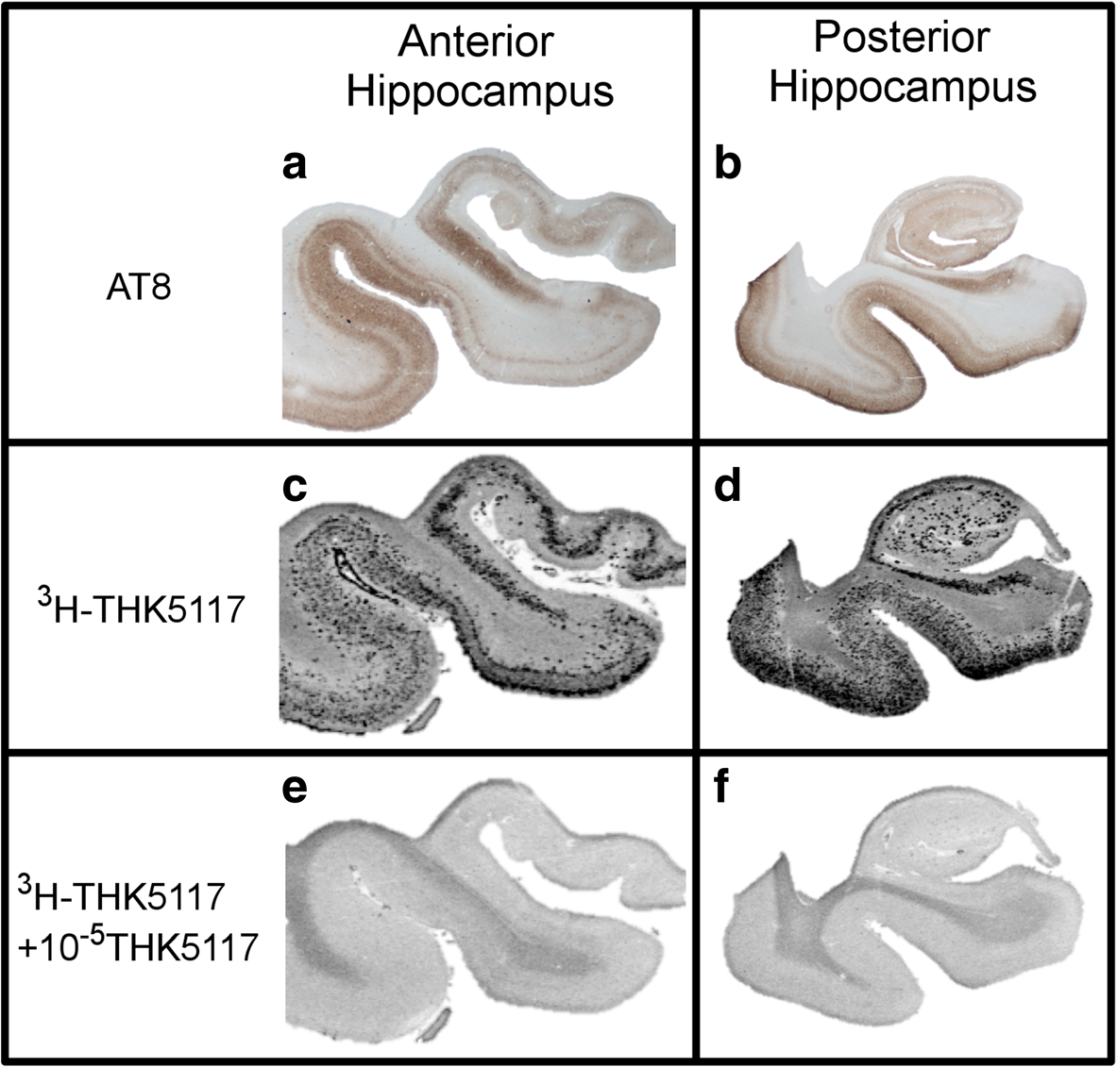
Comparison between AT8 staining (**a-b**) and ^3^H-THK5117 autoradiography **(c-f)** performed on paraffin sections from anterior and posterior hippocampus of AD case one’s right hemisphere. (**c-d**) ^3^H-THK5117 autoradiography, (**e-f**) ^3^H-THK5117 + 10^−5^ M of THK5117 autoradiography for anterior and posterior hippocampus respectively

Autoradiography with ^3^H-THK5117 and immunostaining with AT8 performed on adjacent sections of anterior and posterior part of hippocampus of AD case one showed identical binding pattern (Fig. 4).

**Fig. 5:**
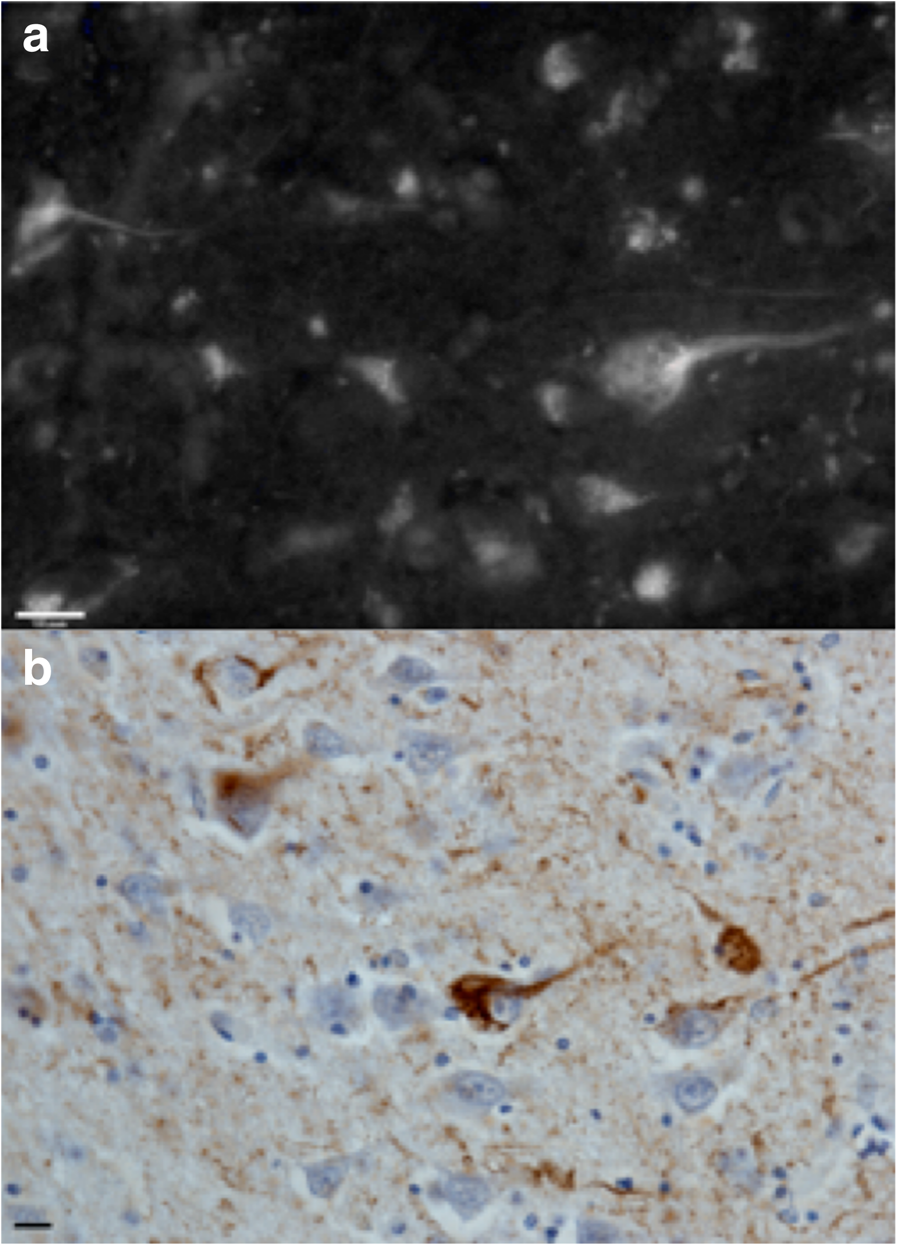
Autofluorescence of THK5117 and AT8 immunostaining on Alzheimer’s disease brain sections. (**a**) Autofluorescence results for THK5117 on paraffin Alzheimer’s disease brain section (bar = 16 μM), (**b**) Immunostaining with AT8 tau antibody on the adjacent section (scale bar = 12 μM)

Autofluorescence performed on the hippocampus section from another AD case revealed THK5117 binding on the neurofibrillary tangles confirmed by tau immunostaining with the AT8 tau antibody on the adjacent section (Fig. 5).

### Correlation of *in vivo*^18^F-FDG PET with *in vitro*^3^H-THK5117 autoradiography binding results

Regional *in vivo*^18^F-FDG PET data were available for all three AD cases. A hypometabolism pattern characteristic of AD was observed in all cases. The inter-rater agreement for manual segmentation was within the 95 % limit of agreement for all defined regions of interest in all patients.

For the three cases, hippocampus showed the lowest ^18^F-FDG PET uptake and one of the highest ^3^H-THK5117 binding results (see Additional file 6 for ^18^F-FDG uptake values).

**Fig. 6:**
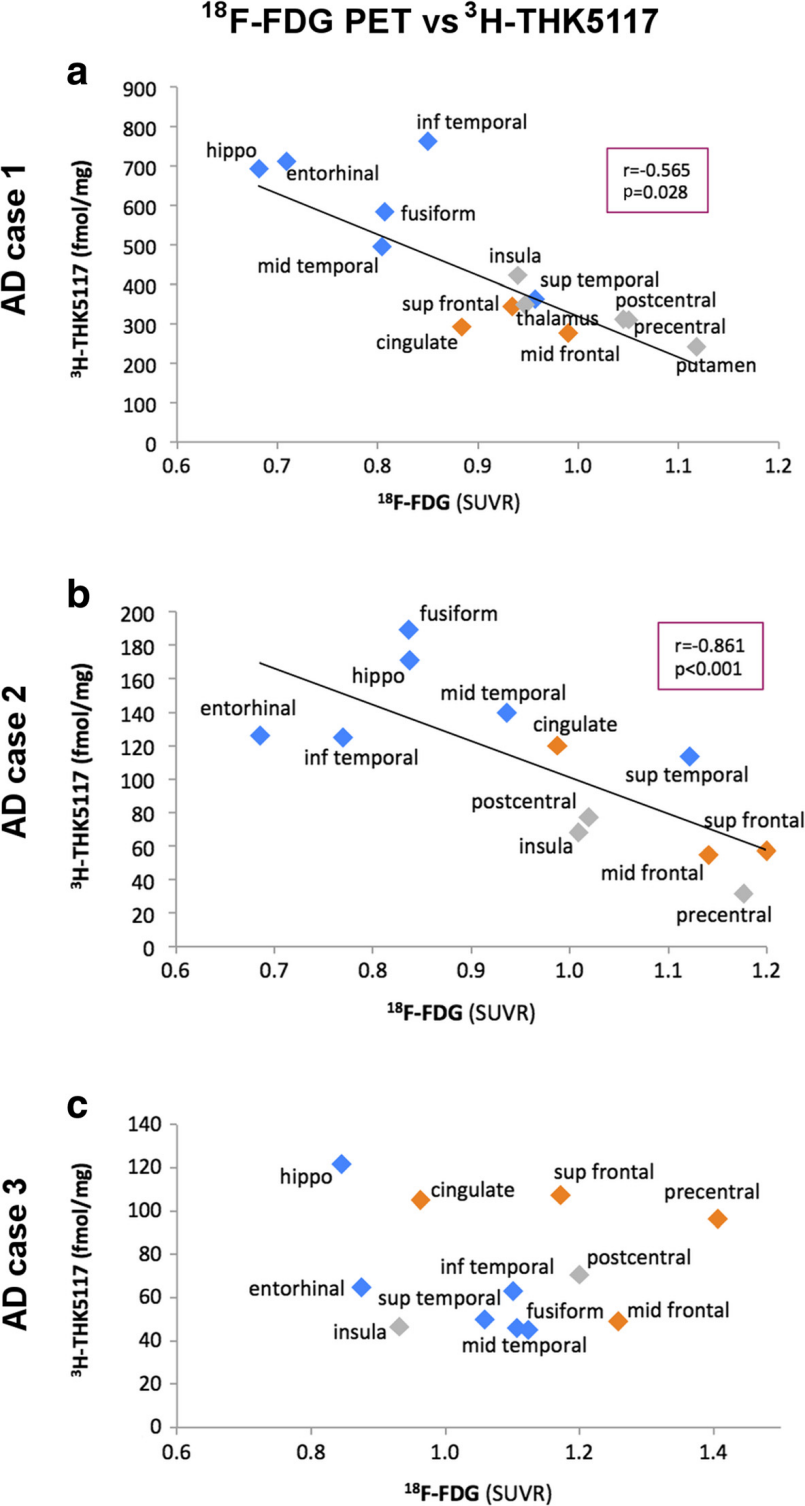
Correlations between *in vitro* ^3^H-THK5117 autoradiography binding and *in vivo* ^18^F-FDG uptake in case one (**a**), case two (**b**), and case three (**c**). All ^18^F-FDG uptake values are reported as standard uptake value ratios (SUVR) with reference to the grey matter of the cerebellum. Temporal regions are shown in blue, frontal regions in orange, the rest in grey. r and p values from the Spearman test are reported for significant correlations (significance threshold: p < 0.05). Regions were defined by manual segmentation. hippo = hippocampus; inf = inferior; mid = middle; sup = superior

A significant negative correlation was found in AD case one and two between *in vivo* uptake of ^18^F-FDG and *in vitro*^3^H-THK5117 autoradiography binding while no significant correlation was observed for AD case three (Fig. 6).

## Discussion

In this study we explored the binding properties and regional distribution of the novel PET tau tracer THK5117 in autopsy brain tissue homogenates and autoradiography on whole frozen brain hemisphere. ^3^H-THK5117 showed a high affinity for tau, as observed in competition binding studies where ^3^H-THK5117 demonstrated up to three binding sites. In saturation binding assays, we could observe two binding sites, one being in the nanomolar range, in agreement with Okamura et al. [17].

Binding assays performed in brain homogenates should reflect the actual binding situation more accurately and are supposed to be more reliable than binding assays performed on tau fibrils, but they also reflect pathological variability between subjects, possibly leading to more heterogeneity in the results.

Since the selectivity of a PET tracer for its target is of crucial importance, THK5117 binding was assessed in competition studies with other compounds. No competition was observed with the amyloid tracers ^3^H-PIB and BTA-1. More interestingly, a trend for competition was observed between ^3^H-THK5117 and FDDNP at the super-high affinity binding site. FDDNP was initially introduced as Aß PET tracer [12, 21] but has then been reported to also bind to NFTs [22, 23], and to correlate with cognitive decline [11]. As suggested previously [24], the THK compounds family (such as THK523 and now THK5117) could share some binding properties with FDDNP; all of them are of interest for targeting tau pathology.

The ^3^H-THK5117 binding in AD tissue homogenates was highest in both the hippocampus and temporal cortex which are regions that are expected to be more severely affected earlier by tau pathology in AD [6, 25]. Consistent findings were also observed in brain hemisphere sections used in autoradiography, with the highest binding in the temporal regions for the three AD cases. Interestingly, those three cases, all with different clinical presentations, differed markedly in regional ^3^H-THK5117 binding intensity.

In AD case one, presenting with an early onset of AD, ^3^H-THK5117 binding in the hippocampus and the entorhinal cortex was about six times higher than in the other two AD cases. This difference could also been observed on AT8 staining for tau, in particular in hippocampus. Indeed case one’s brain was severely affected by the disease, in particular in those temporal regions, and was very shrunken, due to neuronal loss. This atrophy could partly explain the higher extent of binding compared to case two and three, in whom the superficial layers were more compact and dense. One could also assume that the high level of ^3^H-THK5117 binding could be related to the early onset of the disease. It has been suggested that EOAD and LOAD might have different mechanisms for disease progression [26]. Some of the AD patients included in the binding assays also had EOAD. However, there were no differences between EOAD and LOAD in ^3^H-THK5117 binding except in the caudate nucleus homogenates, where it was higher in EOAD tissue than in LOAD tissue. Although tau pathology has been reported in the striatum of both EOAD and LOAD brains [27], no comparisons between the two clinical entities in this respect have been reported to our knowledge. Interestingly, control tissue also showed binding in caudate, that appeared similar to the one observed in LOAD tissue. In recent in vivo Tau PET imaging studies, high signal has been reported in striatum of both controls and AD patients. Although it has been suggested that this binding would be non-specific, no evidence has been provided yet. Both our binding assays and autoradiography in AD case one showed to some extent specific binding in striatum of both control and AD cases. Our limited sample prevents us from drawing any conclusion, and specific investigation addressing this issue should be performed.

Compared to case one and three, case two showed an in-between binding of ^3^H-THK5117 in hippocampus and frontal cortex, which is in agreement with the numerous tau lesions reported on pathological examination. ^3^H-THK5117 binding was lowest in case three, who had only few tangles in the frontal cortex despite a lot of tau in the granular cell layer of the hippocampus. This interesting pattern is in agreement with the clinical presentation of the patient, who showed dramatically pronounced episodic memory impairment, but was less impaired in global cognition than the other two patients. One limitation of this study is the sample size. The *in vitro* investigations need to be performed on a larger sample to address the variability of both clinical and pathological presentation in AD patients. Furthermore, a next step would be to investigate THK5117 binding distributions in other tauopathies. Since tau exists in different forms, it would be important to assess the form of tau to which THK5117 is binding, as this may have implications for our understanding of the different tauopathies.

Autoradiography binding studies with (*S*)-^18^F-THK5117 indicated binding patterns that were very similar to those of ^3^H-THK5117 in all three patients, although autoradiography with tritiated compounds is associated with higher resolution than autoradiography with fluorinated compounds. This finding is very promising for use in *in vivo* PET scans.

One interesting characteristics of this study was that autoradiography was performed on hemisphere sections from three patients with confirmed AD who had previously undergone ^18^F-FDG during the early course of their disease. Thus, it was possible to compare the post-mortem THK5117 binding patterns with the ante-mortem patterns for ^18^F-FDG in the same subjects. Although these findings should be viewed with caution, since the PET scans had been performed several years before death, a negative correlation between *in vivo*^18^F-FDG uptake and post-mortem THK5117 binding was observed in two AD cases. More severe tau pathology is observed in regions with lower cerebral glucose metabolism while less tau pathology is found in regions with preserved metabolism, which may support the assumption that NFTs trigger neuronal dysfunction. No significant correlation was found for case three. Since α-synucleinopathy was reported in this case, although little is known about the relationship between α-synuclein and tau in AD, pathological processes involving α-synuclein may have influenced pathological progression in this patient.

## Conclusions

In conclusion, the tau tracer THK5117 binds with high affinity and good selectivity to cerebral tau lesions. The binding patterns for ^3^H-THK5117 not only showed significant differences between AD patients and controls, but also appeared to reflect the different tau pathology as well as clinical symptoms of the patients. The use of different *in vivo* tau tracers in PET imaging will shed further light on the role of tau pathology in the time course of AD and its concurrent clinical features.

## Additional files

Additional file 1:
**Immunostaining of AD case one, two and three using AT8 and Clone 6 F/3D staining in Frontal cortex (A-F) and Hippocampus (G-L).** **J’,K’,L’** show a zoom in the granular cell layer of the dentate gyrus. All images are magnification 4x, scale bar = 300 μM except for **J’, K’**and **L’** magnification 20x scale bar = 100 μM. AD *case one*: Numerous NFTs were seen throughout the cortex in AT8 staining prominently in superficial layers (D). Tangles were also found in many of the remaining hippocampal nerve cells, and intracytoplasmic positivity was seen in several granular cells of the dentate gyrus (J, J’). Staining with clone 6F/3D showed large numbers of Ab plaques in the cortex, many with a central core (A), while fewer were found in the hippocampus (G).AD *case two*: AT8 staining indicated numerous NFTs in the frontal cortex (E). In the granular cells, only a few neurons stained positively for tau (K, K’). Numerous Ab plaques were seen in the cortex (B), some of which had a core, while there were only a few plaques in the hippocampus (H). Staining of vessels in the cortex and leptomeninges was extensive.AD *case three*: AT8 positivity was found in relatively few cortical neurons prominently in superficial layers as for case one (F), and a peculiar pattern was seen in the granular cell layer of the dentate gyrus with prominent intracytoplasmic staining (L, L’). Staining with clone 6F/3D revealed extensive amyloid in the cortical vessels, but very few cortical plaques (C). Additional file 2:
**A. Saturation binding curve and B. Scatchard plot of** ^**3**^ **H-THK5117 (0.04-270nM) for hippocampus homogenates of one AD patient.** The plain regression line was determined by GraphPad Prism software and corresponds to the low affinity site. The dotted regression line was determined manually and corresponds to the high affinity site. Corresponding Kd and Bmax values are mentioned below the curve. **C**. Competition binding curve between THK5117 (10^-13^-10^-5^ M) and ^3^H-THK5117 (3nM) for hippocampus homogenates of one AD case (75 years old). Analyses from non-linear regression using a least square ordinary fit in GraphPad Prism software show two different binding sites. Ki Sup Hi = Ki for super high binding site; Ki Hi = Ki for high binding site; Ki Lo = Ki for low binding site. Additional file 3:
**Competition binding curve for temporal cortex homogenates of four AD cases between** ^**3**^ **H-THK5117 (3nM) and A. unlabeled FDDNP (10** ^**-6**^ **-10** ^**-14**^ **M); B. unlabeled BTA-1 (10** ^**-5**^ **-10** ^**-13**^ **M).** Analyses from non-linear regression using a least square ordinary fit in GraphPad Prism software show no common binding site. Ki: inhibitory constant. r^2^ = regression coefficient. "Ambiguous" is a term coined by GraphPad to describe a fit that doesn't really nail down the values of all the parameters (extract from GraphPad Curve Fitting Guide). Additional file 4:
**Autoradiography in AD case two and AD case three. A-B Autoradiography of adjacent left hemisphere sections from case two.** **A**. Autoradiography with ^3^H-THK5117. **B.** Autoradiography with ^3^H-THK5117 + 10^-5^ M of THK5117. **C-D** Autoradiography of adjacent left hemisphere sections from case three. **C**. Autoradiography with ^3^H-THK5117. **D**. Autoradiography with ^3^H-THK5117 + 10^-5^ M of THK5117. Additional file 5:
**Autoradiography results from adjacent left hemisphere sections from the three AD cases with (** ***S*** **)-** ^**18**^ **F-THK5117.**
Additional file 6:
**Regional standard uptake values for** ^**18**^ **F-FDG PET scans in threeAD cases.** Regions of interest were defined for each case by manual segmentation of a coronal section of the individual’s MR image (case two & three) or an MR template (case one). Segmentations were performed on the left hemisphere in order to match the autoradiography results. Values are expressed as Standard uptake values with cerebellum uptake as reference (SUVR). NA = not available.

## References

[1] Nordberg A (2010). Amyloid imaging in early detection of Alzheimer’s disease. Neurodegener Dis.

[2] Seppala TT, Nerg O, Koivisto AM, Rummukainen J, Puli L, Zetterberg H, Pyykko OT, Helisalmi S, Alafuzoff I, Hiltunen M (2012). CSF biomarkers for Alzheimer disease correlate with cortical brain biopsy findings. Neurology.

[3] Strozyk D, Blennow K, White LR, Launer LJ (2003). CSF Abeta 42 levels correlate with amyloid-neuropathology in a population-based autopsy study. Neurology.

[4] Zetterberg H, Lautner R, Skillback T, Rosen C, Shahim P, Mattsson N, Blennow K (2014). CSF in Alzheimer’s disease. Adv Clin Chem.

[5] Villemagne VL, Fodero-Tavoletti MT, Masters CL, Rowe CC (2015). Tau imaging: early progress and future directions. The Lancet Neurology.

[6] Arriagada PV, Growdon JH, Hedley-Whyte ET, Hyman BT (1992). Neurofibrillary tangles but not senile plaques parallel duration and severity of Alzheimer’s disease. Neurology.

[7] Bierer LM, Hof PR, Purohit DP, Carlin L, Schmeidler J, Davis KL, Perl DP (1995). Neocortical neurofibrillary tangles correlate with dementia severity in Alzheimer’s disease. Arch Neurol.

[8] Giannakopoulos P, Herrmann FR, Bussiere T, Bouras C, Kovari E, Perl DP, Morrison JH, Gold G, Hof PR (2003). Tangle and neuron numbers, but not amyloid load, predict cognitive status in Alzheimer’s disease. Neurology.

[9] Naslund J, Haroutunian V, Mohs R, Davis KL, Davies P, Greengard P, Buxbaum JD (2000). Correlation between elevated levels of amyloid beta-peptide in the brain and cognitive decline. JAMA.

[10] Agdeppa ED, Kepe V, Liu J, Flores-Torres S, Satyamurthy N, Petric A, Cole GM, Small GW, Huang SC, Barrio JR (2001). Binding characteristics of radiofluorinated 6-dialkylamino-2-naphthylethylidene derivatives as positron emission tomography imaging probes for beta-amyloid plaques in Alzheimer’s disease. J Neurosci.

[11] Tolboom N, van der Flier WM, Yaqub M, Koene T, Boellaard R, Windhorst AD, Scheltens P, Lammertsma AA, van Berckel BN (2009). Differential association of [11C]PIB and [^18^F]FDDNP binding with cognitive impairment. Neurology.

[12] Thompson PW, Ye L, Morgenstern JL, Sue L, Beach TG, Judd DJ, Shipley NJ, Libri V, Lockhart A (2009). Interaction of the amyloid imaging tracer FDDNP with hallmark Alzheimer’s disease pathologies. J Neurochem.

[13] Okamura N, Harada R, Furumoto S, Arai H, Yanai K, Kudo Y (2014). Tau PET imaging in Alzheimer’s disease. Curr Neurol Neurosci Rep.

[14] Chien DT, Szardenings AK, Bahri S, Walsh JC, Mu F, Xia C, Shankle WR, Lerner AJ, Su MY, Elizarov A (2014). Early clinical PET imaging results with the novel PHF-tau radioligand [F18]-T808. J Alzheimers Dis.

[15] Harada R, Okamura N, Furumoto S, Furukawa K, Ishiki A, Tomita N, Hiraoka K, Watanuki S, Shidahara M, Miyake M (2015). [(18)F]THK-5117 PET for assessing neurofibrillary pathology in Alzheimer’s disease. Eur J Nucl Med Mol Imaging.

[16] Maruyama M, Shimada H, Suhara T, Shinotoh H, Ji B, Maeda J, Zhang MR, Trojanowski JQ, Lee VM, Ono M (2013). Imaging of tau pathology in a tauopathy mouse model and in Alzheimer patients compared to normal controls. Neuron.

[17] Okamura N, Furumoto S, Harada R, Tago T, Yoshikawa T, Fodero-Tavoletti M, Mulligan RS, Villemagne VL, Akatsu H, Yamamoto T (2013). Novel ^18^F-labeled arylquinoline derivatives for noninvasive imaging of tau pathology in Alzheimer disease. J Nucl Med.

[18] Gillberg PG, Jossan SS, Askmark H, Aquilonius SM (1986). Large-section cryomicrotomy for in vitro receptor autoradiography. J Pharmacol Methods.

[19] Duvernoy HM (2005). The Human Hippocampus.

[20] Okamura N, Suemoto T, Shimadzu H, Suzuki M, Shiomitsu T, Akatsu H, Yamamoto T, Staufenbiel M, Yanai K, Arai H (2004). Styrylbenzoxazole derivatives for in vivo imaging of amyloid plaques in the brain. J Neurosci.

[21] Shin J, Kepe V, Barrio JR, Small GW (2011). The merits of FDDNP-PET imaging in Alzheimer’s disease. Journal of Alzheimer’s disease: JAD.

[22] Barrio JR, Kepe V, Satyamurthy N, Huang SC, Small G (2008). Amyloid and tau imaging, neuronal losses and function in mild cognitive impairment. J Nutr Health Aging.

[23] Shoghi-Jadid K, Small GW, Agdeppa ED, Kepe V, Ercoli LM, Siddarth P, Read S, Satyamurthy N, Petric A, Huang SC (2002). Localization of neurofibrillary tangles and beta-amyloid plaques in the brains of living patients with Alzheimer disease. Am J Geriatr Psychiatry.

[24] Harada R, Okamura N, Furumoto S, Tago T, Maruyama M, Higuchi M, Yoshikawa T, Arai H, Iwata R, Kudo Y (2013). Comparison of the binding characteristics of [^18^F]THK-523 and other amyloid imaging tracers to Alzheimer’s disease pathology. Eur J Nucl Med Mol Imaging.

[25] Braak H, Braak E (1991). Neuropathological stageing of Alzheimer-related changes. Acta Neuropathol.

[26] Shinohara M, Fujioka S, Murray ME, Wojtas A, Baker M, Rovelet-Lecrux A, Rademakers R, Das P, Parisi JE, Graff-Radford NR (2014). Regional distribution of synaptic markers and APP correlate with distinct clinicopathological features in sporadic and familial Alzheimer’s disease. Brain.

[27] Braak H, Braak E (1990). Alzheimer’s disease: striatal amyloid deposits and neurofibrillary changes. J Neuropathol Exp Neurol.

