# Supplementary material for: Visualization of regional tau deposits using 3H-THK5117 in Alzheimer brain tissue
Source: Acta Neuropathol Commun. 2015 Jul 2;3:40. doi: 10.1186/s40478-015-0220-4 (PMC4489196; doi:10.1186/s40478-015-0220-4)
Supplement: Additional file 1: — Immunostaining of AD case one, two and three using AT8 and Clone 6 F/3D staining in Frontal cortex (A-F) and Hippocampus (G-L). J’,K’,L’ show a zoom in the granular cell layer of the dentate gyrus. All images are magnification 4x, scale bar = 300 μM except for J’, K’and L’ magnification 20x scale bar = 100 μM. AD case one: Numerous NFTs were seen throughout the cortex in AT8 staining prominently in superficial layers (D). Tangles were also found in many of the remaining hippocampal nerve cells, and intracytoplasmic positivity was seen in several granular cells of the dentate gyrus (J, J’). Staining with clone 6F/3D showed large numbers of Ab plaques in the cortex, many with a central core (A), while fewer were found in the hippocampus (G).AD case two: AT8 staining indicated numerous NFTs in the frontal cortex (E). In the granular cells, only a few neurons stained positively for tau (K, K’). Numerous Ab plaques were seen in the cortex (B), some of which had a core, while there were only a few plaques in the hippocampus (H). Staining of vessels in the cortex and leptomeninges was extensive.AD case three: AT8 positivity was found in relatively few cortical neurons prominently in superficial layers as for case one (F), and a peculiar pattern was seen in the granular cell layer of the dentate gyrus with prominent intracytoplasmic staining (L, L’). Staining with clone 6F/3D revealed extensive amyloid in the cortical vessels, but very few cortical plaques (C). [file 40478_2015_220_MOESM1_ESM.doc]

**Additional file 1**

Immunostaining of AD case one, two and three using AT8 and Clone 6F/3D staining in Frontal cortex (**A-F**) and Hippocampus (**G-L**). **J’,K’,L’** show a zoom in the granular cell layer of the dentate gyrus. All images are magnification 4x, scale bar= 300 μM except for **J’, K’**and **L’** magnification 20x scale bar=100 μM**.**

*AD case one:* Numerous NFTs were seen throughout the cortex in AT8 staining prominently in superficial layers (**D**). Tangles were also found in many of the remaining hippocampal nerve cells, and intracytoplasmic positivity was seen in several granular cells of the dentate gyrus (**J, J’**). Staining with clone 6F/3D showed large numbers of Aβ plaques in the cortex, many with a central core (**A**), while fewer were found in the hippocampus (**G**).

*AD case two:* AT8 staining indicated numerous NFTs in the frontal cortex (**E**). In the granular cells, only a few neurons stained positively for tau (**K, K’**). Numerous Aβ plaques were seen in the cortex (**B**), some of which had a core, while there were only a few plaques in the hippocampus (**H**). Staining of vessels in the cortex and leptomeninges was extensive.

*AD case three:* AT8 positivity was found in relatively few cortical neurons prominently in superficial layers as for case one (**F**), and a peculiar pattern was seen in the granular cell layer of the dentate gyrus with prominent intracytoplasmic staining (**L, L’**). Staining with clone 6F/3D revealed extensive amyloid in the cortical vessels, but very few cortical plaques (**C**).

**
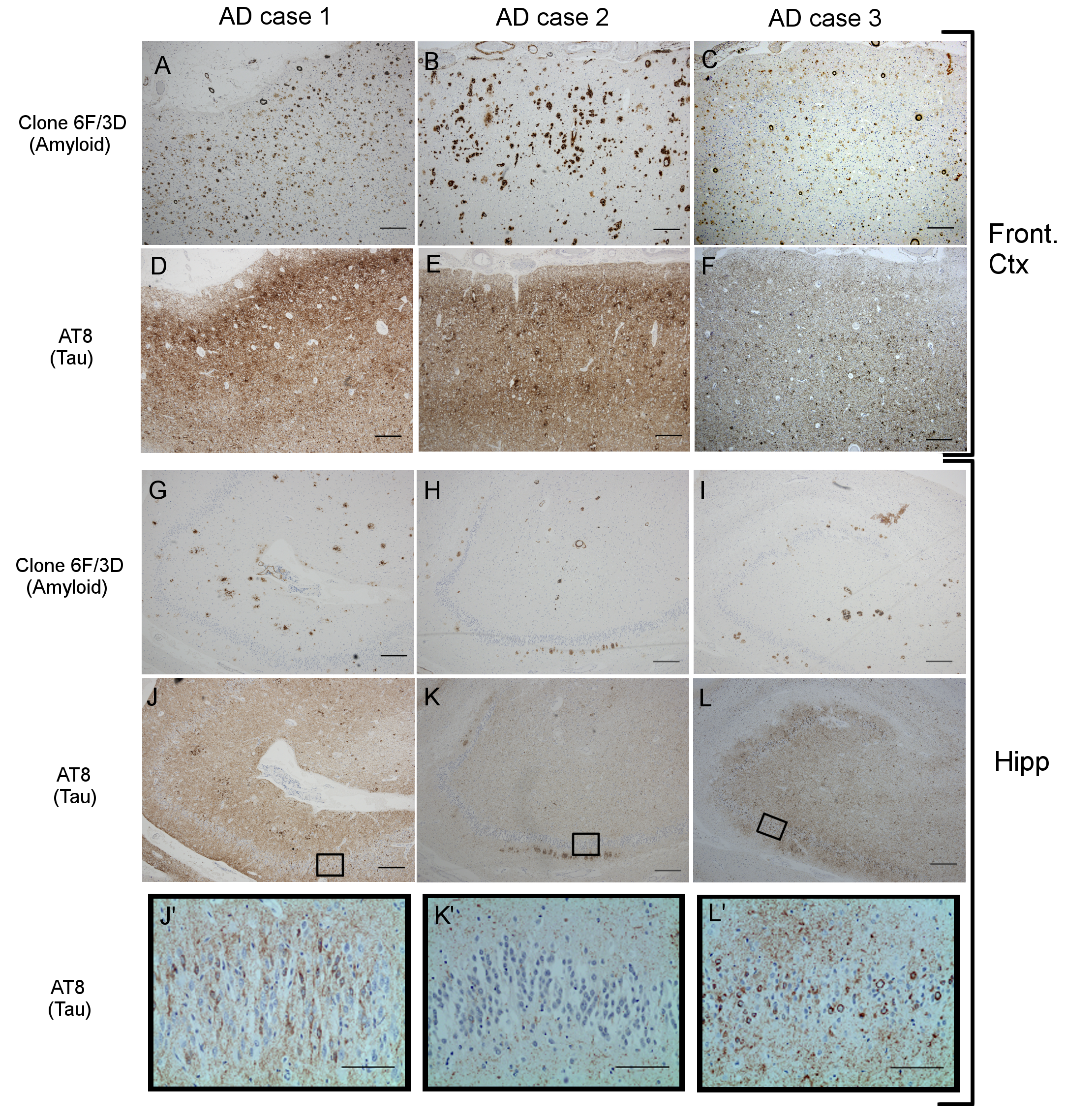
**
