# Supplementary material for: Visualization of regional tau deposits using 3H-THK5117 in Alzheimer brain tissue
Source: Acta Neuropathol Commun. 2015 Jul 2;3:40. doi: 10.1186/s40478-015-0220-4 (PMC4489196; doi:10.1186/s40478-015-0220-4)

**Additional file 2**

**A**. Saturation binding curve and **B**. Scatchard plot of ^3^H-THK5117 (0.04-270nM) for hippocampus homogenates of one AD patient. The plain regression line was determined by GraphPad Prism software and corresponds to the low affinity site. The dotted regression line was determined manually and corresponds to the high affinity site. Corresponding Kd and Bmax values are mentioned below the curve. **C**. Competition binding curve between THK5117 (10^-13^-10^-5^M) and ^3^H-THK5117 (3nM) for hippocampus homogenates of one AD case (75 years old). Analyses from non-linear regression using a least square ordinary fit in GraphPad Prism software show two different binding sites. Ki Sup Hi = Ki for super high binding site; Ki Hi = Ki for high binding site; Ki Lo = Ki for low binding site.


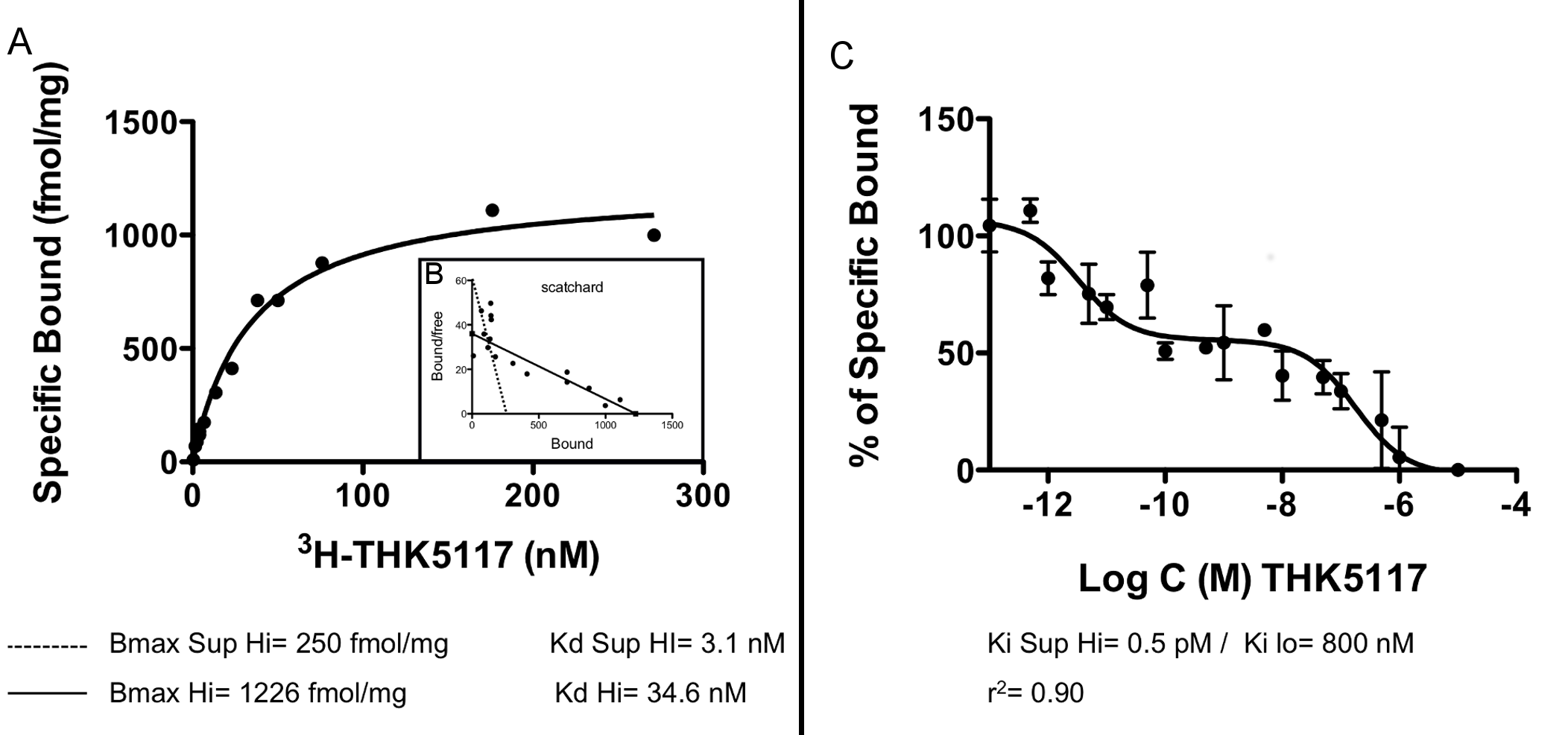

Supplement: Additional file 2: — A. Saturation binding curve and B. Scatchard plot of 3 H-THK5117 (0.04-270nM) for hippocampus homogenates of one AD patient. The plain regression line was determined by GraphPad Prism software and corresponds to the low affinity site. The dotted regression line was determined manually and corresponds to the high affinity site. Corresponding Kd and Bmax values are mentioned below the curve. C. Competition binding curve between THK5117 (10-13-10-5 M) and 3H-THK5117 (3nM) for hippocampus homogenates of one AD case (75 years old). Analyses from non-linear regression using a least square ordinary fit in GraphPad Prism software show two different binding sites. Ki Sup Hi = Ki for super high binding site; Ki Hi = Ki for high binding site; Ki Lo = Ki for low binding site. [file 40478_2015_220_MOESM2_ESM.doc]
