# Supplementary material for: Visualization of regional tau deposits using 3H-THK5117 in Alzheimer brain tissue
Source: Acta Neuropathol Commun. 2015 Jul 2;3:40. doi: 10.1186/s40478-015-0220-4 (PMC4489196; doi:10.1186/s40478-015-0220-4)
Supplement: Additional file 3: — Competition binding curve for temporal cortex homogenates of four AD cases between 3 H-THK5117 (3nM) and A. unlabeled FDDNP (10 -6 -10 -14 M); B. unlabeled BTA-1 (10 -5 -10 -13 M). Analyses from non-linear regression using a least square ordinary fit in GraphPad Prism software show no common binding site. Ki: inhibitory constant. r2 = regression coefficient. "Ambiguous" is a term coined by GraphPad to describe a fit that doesn't really nail down the values of all the parameters (extract from GraphPad Curve Fitting Guide). [file 40478_2015_220_MOESM3_ESM.doc]

**Additional file 3**

Competition binding curve for temporal cortex homogenates of four AD cases between ^3^H-THK5117 (3nM) and A. unlabeled FDDNP (10^-6^-10^-14^M); B. unlabeled BTA-1 (10^-5^-10^-13^ M). Analyses from non-linear regression using a least square ordinary fit in GraphPad Prism software show no common binding site. Ki: inhibitory constant. r^2^= regression coefficient.

"Ambiguous" is a term coined by GraphPad to describe a fit that doesn't really nail down the values of all the parameters (extract from GraphPad Curve Fitting Guide).

**
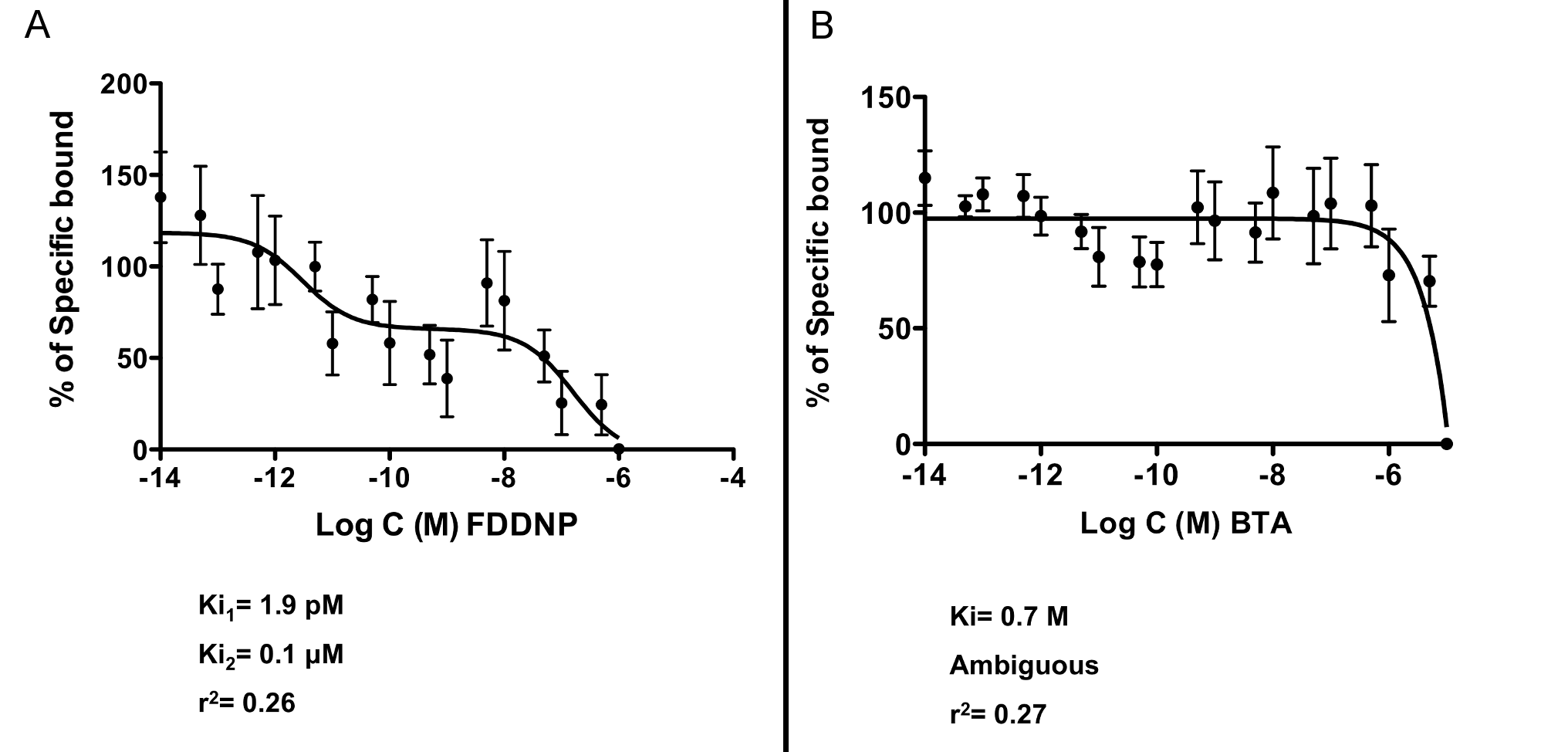
**
