# Supplementary material for: Visualization of regional tau deposits using 3H-THK5117 in Alzheimer brain tissue
Source: Acta Neuropathol Commun. 2015 Jul 2;3:40. doi: 10.1186/s40478-015-0220-4 (PMC4489196; doi:10.1186/s40478-015-0220-4)

**Additional file 4**

Autoradiography in AD case two and AD case three. **A-B** Autoradiography of adjacent left hemisphere sections from case two. **A**. Autoradiography with ^3^H-THK5117. **B.** Autoradiography with ^3^H-THK5117 + 10^-5^M of THK5117. **C-D** Autoradiography of adjacent left hemisphere sections from case three. **C**. Autoradiography with ^3^H-THK5117. **D**. Autoradiography with ^3^H-THK5117 + 10^-5^M of THK5117.


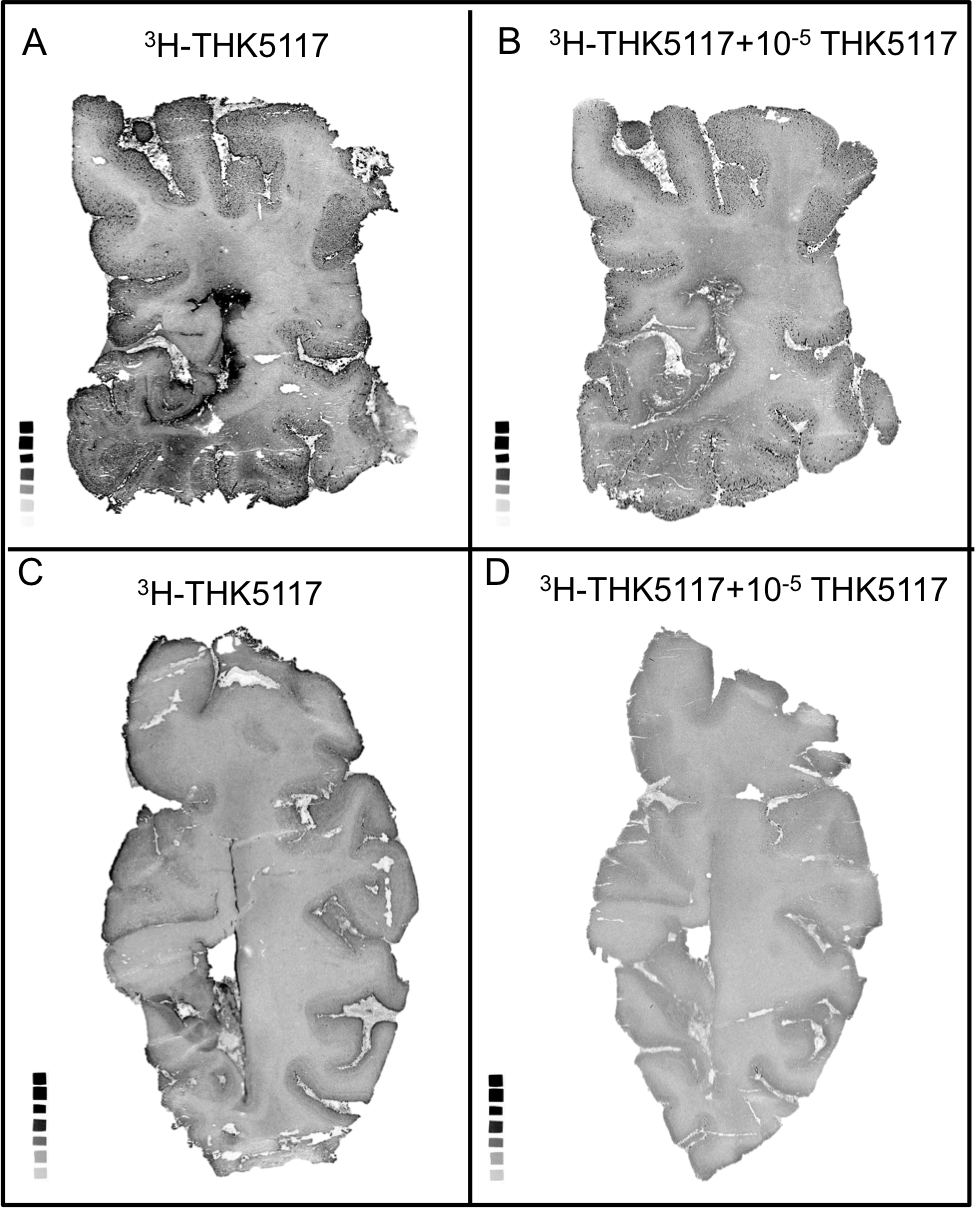

Supplement: Additional file 4: — Autoradiography in AD case two and AD case three. A-B Autoradiography of adjacent left hemisphere sections from case two. A. Autoradiography with 3H-THK5117. B. Autoradiography with 3H-THK5117 + 10-5 M of THK5117. C-D Autoradiography of adjacent left hemisphere sections from case three. C. Autoradiography with 3H-THK5117. D. Autoradiography with 3H-THK5117 + 10-5 M of THK5117. [file 40478_2015_220_MOESM4_ESM.doc]
