# Supplementary material for: Visualization of regional tau deposits using 3H-THK5117 in Alzheimer brain tissue
Source: Acta Neuropathol Commun. 2015 Jul 2;3:40. doi: 10.1186/s40478-015-0220-4 (PMC4489196; doi:10.1186/s40478-015-0220-4)
Supplement: Additional file 5: — Autoradiography results from adjacent left hemisphere sections from the three AD cases with ( S )- 18 F-THK5117. [file 40478_2015_220_MOESM5_ESM.doc]

**Additional file 5**

Autoradiography results from adjacent left hemisphere sections from the three AD cases with (*S*)-^18^F-THK5117

**
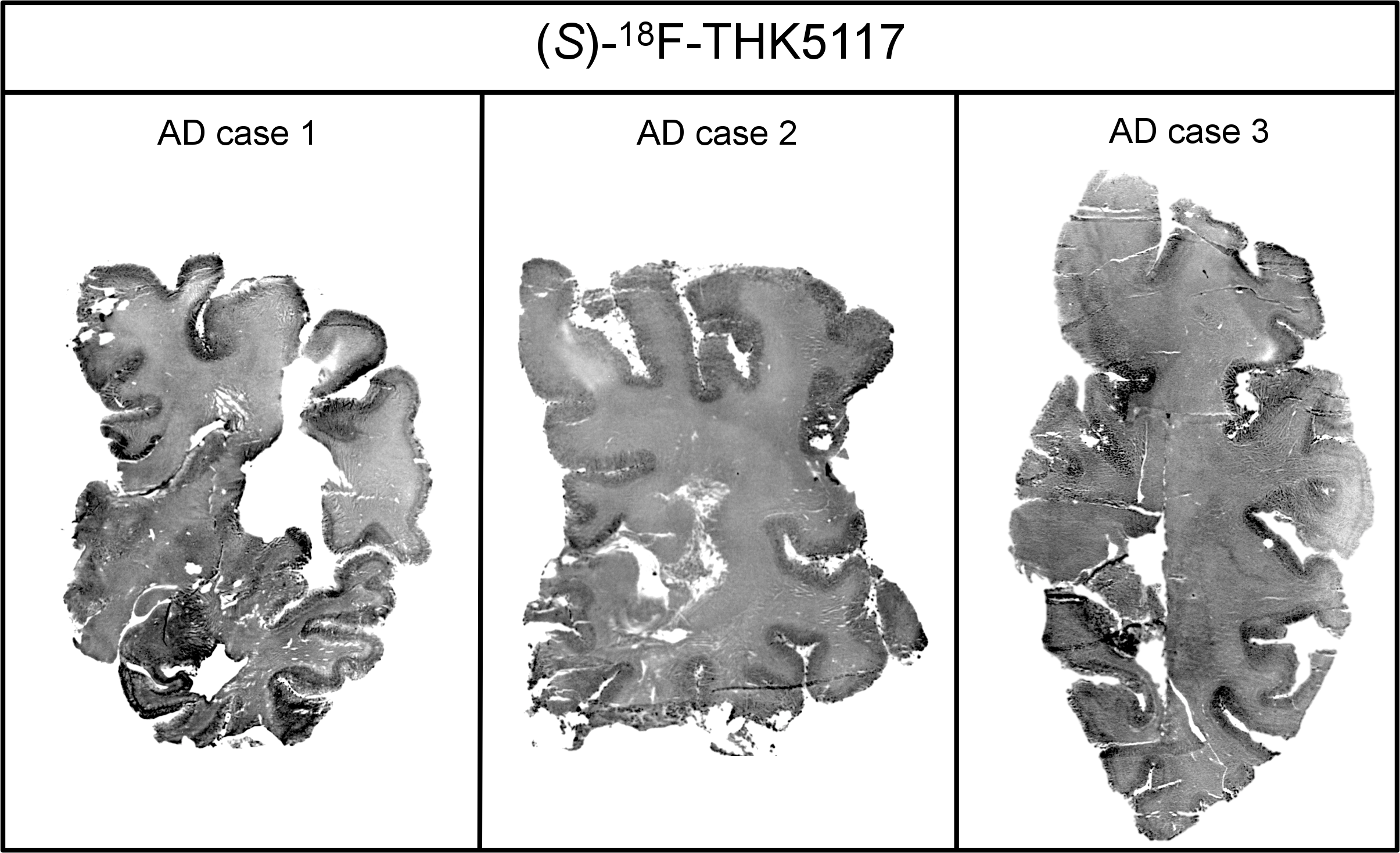
**
