# Supplementary material for: Visualization of regional tau deposits using 3H-THK5117 in Alzheimer brain tissue
Source: Acta Neuropathol Commun. 2015 Jul 2;3:40. doi: 10.1186/s40478-015-0220-4 (PMC4489196; doi:10.1186/s40478-015-0220-4)
Supplement: Additional file 6: — Regional standard uptake values for 18 F-FDG PET scans in threeAD cases. Regions of interest were defined for each case by manual segmentation of a coronal section of the individual’s MR image (case two & three) or an MR template (case one). Segmentations were performed on the left hemisphere in order to match the autoradiography results. Values are expressed as Standard uptake values with cerebellum uptake as reference (SUVR). NA = not available. [file 40478_2015_220_MOESM6_ESM.doc]

**Additional file 6**

Regional standard uptake values for ^18^F-FDG PET scans in threeAD cases. Regions of interest were defined for each case by manual segmentation of a coronal section of the individual’s MR image (case two & three) or an MR template (case one). Segmentations were performed on the left hemisphere in order to match the autoradiography results. Values are expressed as Standard uptake values with cerebellum uptake as reference (SUVR). NA=not available.

|  | FDG (SUVR) | | | | | | | | |
| --- | --- | --- | --- | --- | --- | --- | --- | --- | --- |
|  | AD case 1 | | AD case 2 | | | | AD case 3 | | |
|  | baseline | follow-up | baseline | follow-up 1 | follow-up 2 | follow-up 3 | baseline | follow-up 1 | follow-up 2 |
| years before death | 9 | 7 | 8 | 8 | 8 | 8 | 10 | 9 | 5 |
| Hippocampus | 0.68 | 0.63 | 0.85 | 0.83 | 0.71 | 0.82 | 0.88 | 0.87 | 0.66 |
| Entorhinal cortex | 0.72 | 0.80 | 0.75 | 0.84 | 0.63 | 0.68 | 0.89 | 0.88 | 0.68 |
| Fusiform gyrus | 0.81 | 0.90 | 0.83 | 0.88 | 0.75 | 0.83 | 1.13 | 1.03 | 0.84 |
| Inferior temporal gyrus | 0.85 | 0.84 | 0.75 | 0.73 | 0.61 | 0.73 | 1.10 | 1.00 | 0.80 |
| Middle temporal gyrus | 0.79 | 0.67 | 0.91 | 0.91 | 0.86 | 0.87 | 1.09 | 1.00 | 0.82 |
| Superior temporal gyrus | 0.96 | 0.76 | 1.11 | 1.09 | 0.97 | 1.03 | 1.05 | 1.01 | 0.89 |
| Insular cortex | 0.94 | 0.91 | 1.01 | 1.10 | 1.04 | 1.03 | 0.91 | 0.84 | 1.03 |
| Postcentral gyrus | 1.04 | 0.92 | 1.03 | 1.02 | 0.95 | 1.03 | 1.19 | 1.15 | 1.14 |
| Precentral gyrus | 1.05 | 1.04 | 1.23 | 1.25 | 1.21 | 1.20 | 1.42 | 1.40 | 1.30 |
| Middle frontal gyrus | 1.01 | 1.07 | 1.12 | 1.13 | 1.14 | 1.19 | 1.27 | 1.26 | 1.19 |
| Superior frontal gyrus | 0.98 | 1.14 | 1.21 | 1.21 | 1.11 | 1.24 | 1.17 | 1.17 | 1.15 |
| Cingulate gyrus | 0.88 | 0.81 | 1.00 | 1.08 | 0.99 | 1.07 | 0.97 | 1.11 | 1.13 |
| Putamen | 1.12 | 1.02 | NA | NA | NA | NA | NA | NA | NA |
